# Closed Reduction of Bilateral Posterior Shoulder Dislocation with Medium Impression Defect of the Humeral Head: A Case Report and Review of Its Treatment

**DOI:** 10.1155/2011/124581

**Published:** 2011-11-16

**Authors:** Soorena Rezazadeh, Amir Reza Vosoughi

**Affiliations:** Research Center for Bone & Joint Diseases, Chamran Hospital, Shiraz University of Medical Sciences, Shiraz 7194815644, Iran

## Abstract

Bilateral dislocation of the shoulder is a rare injury. The main causes are electrical shock, extreme trauma, and epilepsy. A 25-year-old athletic-body man had sustained bilateral shoulder pain and restricted external rotation following electrical shock for five days. Although articular surface damage was about 50% in the right side and 30% in the left, it could be managed successfully by close reduction without pinning. During one-year follow-up, no recurrent dislocation or limitation of motion was seen. Closed management of medium size defect of the humeral head after posterior dislocation can be performed in cooperative and especially muscular patients.

## 1. Introduction

Pure posterior dislocation of the shoulder, a commonly missed injury, accounts for about 1 to 4.7% of all shoulder dislocations. Triple “E” syndrome shows three major causes of this entity, epilepsy, electrical shock, and extreme trauma [1]. Bilateral posterior shoulder dislocation, seen in 5% of all posterior dislocations, is the result of seizure attack in 50% of cases. Convulsive seizure will be raised to 90% of etiologies if fracture occurs concomitantly [2–4]. Electrical shock accounts for less than 5% of bilateral posterior shoulder dislocation [1].

Dislocation duration and size of the articular head defect are the major factors in determining treatment plan [5–8]. Acute dislocation (less than 3 weeks from the injury) and small defect up to 25% of the humeral head articular surface can be treated by closed or open reduction [5, 6]. The presented case is the first with bilateral medium size articular defect (25% to 50%) which was treated by closed reduction and casting.

## 2. Case Report

**Figure 1:**
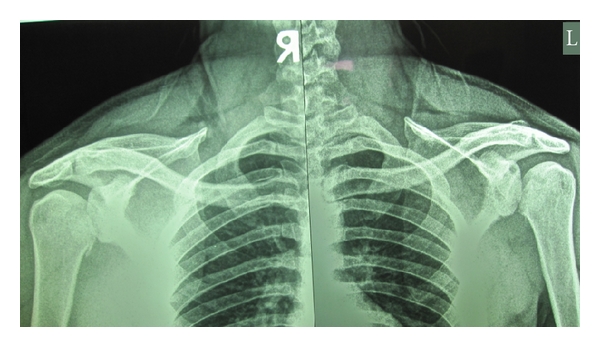
Anteroposterior X-ray shows bilateral posterior dislocation difficulty with absence of normal half-moon sign.

**Figure 2:**
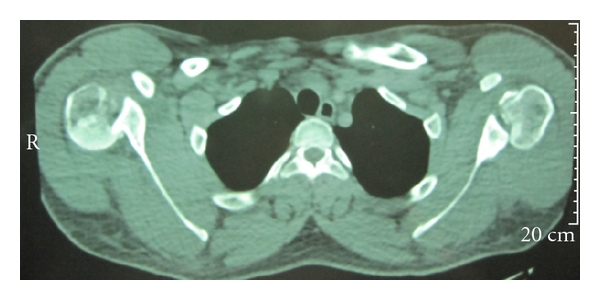
Axial CT scan view reveals bilateral shoulder posterior dislocation with medium defect of articular surfaces (50% in the right, 30% in the left).

A 25-year-old athletic-body gentleman presented with significant pain in both shoulders and inability to do daily activities for 5 days after electrical shock. Immediately after electricity-induced trauma, he had been transferred to a major trauma center. Primary cares had been given. He had been visited by an emergency medicine physician. Routine anteroposterior radiograph of the shoulders (Figure 1) was misinterpreted. Then, he had been discharged with analgesic and arm sling. Five days later, he referred to the senior author with pain and restriction of bilateral shoulder movements. In physical exam, posterior positions of the humeral heads were not palpable due to his muscular body. The patient had fixed both upper limbs in adduction and internal rotation. Passive and active external rotation was blocked and very painful. Moreover, the patient did not permit passive abduction and forward flexion of more than 45 degrees. Neurovascular functions of both sides were normal. Bilateral shoulder posterior dislocation was suspected and CT scan was requested (Figure 2). It showed bilateral posterior dislocation associated with articular surface defect of 50% in the right side and 30% in the left. He underwent closed reduction under general anesthesia after taking consent. Left side reduction was stable but the right was unstable in internal rotation. Bilateral shoulder spica cast was applied in 20 degrees of abduction and 15 degrees of external rotation.

**Figure 3:**
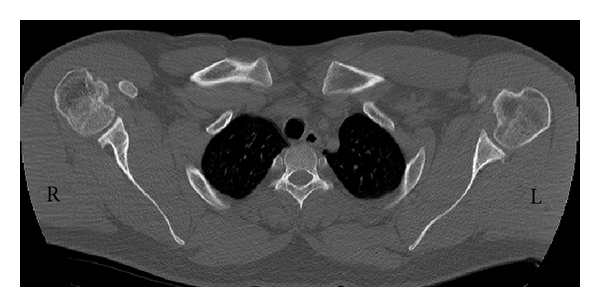
One-year follow-up axial CT scan view shows bilateral small defect (less than 25%) in anteromedial part of the humeral head. These defects are less than what was seen in Figure 2.

After six weeks, the cast was discarded. Stability of joints was well. Rehabilitation program including active and passive range of motion and deltoid and rotator cuff strengthening exercises were begun. As he was a professional athlete in weight lifting, he continued exercises for one year. After one-year follow-up, he had bilateral stable joint with full range of motion without any history of dislocation. CT scan (Figure 3) showed bilateral small defect in anteromedial part of the humeral heads which is more in the left side.

## 3. Discussion

Posterior shoulder dislocation is a rare injury due to very strong soft tissues behind the joint. Most are seen following tonic-clonic seizures [3, 4, 6, 9]. Electrical shock and trauma especially in emotionally disabled patients are the other causes [10]. Posterior shoulder dislocations are common unrecognized injury. Hawkins et al. explained delay in diagnosis by an average of one year in 75% of cases. One of the main reasons is lack of taking axillary or lateral “Y” scapular views. Also they showed true diagnosis in all patients with axillary view [11]. Although several findings including light bulb sign, rim sign, trough line sign, absence of normal half-moon sign, vacant glenoid sign, and Mouzopoulos sign in anteroposterior view were described, usually no one is seen [5, 12]. Moreover, the axillary view is difficult to take due to painful abduction position. So, CT scan is a useful modality, not only for recognizing the posterior dislocation but also for determining the size of articular surface defect and associated fractures [13].

**Figure 4:**
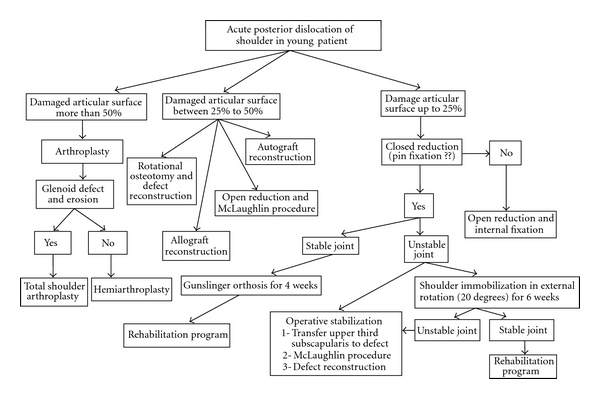
Algorithm of management of acute posterior shoulder dislocation in young patient.

Treatment depends on patient age, duration of dislocation, extent of damage to articular surface, and patient demands and level of activity [5–7, 14, 15]. In acute cases (less than 3 weeks), closed reduction should be attempted if the articular surface defect is small (up to 25%). Medium size defect of 25% to 50% usually needs reconstruction, lesser tuberosity transfer, or rotational osteotomy. Large defect (more than 50%) should be managed by shoulder arthroplasty as described in the algorithm of Figure 4 [6, 11, 15–17].

The presented case had articular impression defect of 50% in the right humeral head and 30% in the left side. We decided to reduce it closely, because he was a muscular cooperative young man. He vigorously continued strengthening exercises of the shoulder girdle muscles during follow-up period. He did not have any history of instability or limitation of motion.

Treatment of patients with delayed diagnosis (more than 3 weeks) depends on viability of humeral head, demand of patient, other comorbidities, and duration of dislocation. If head osteonecrosis is seen or it is diagnosed after 6 months, shoulder replacement is the modality of choice [16–18]. When the head is viable, open reduction and soft tissue release are logical. Other procedures such as humeral osteotomy, McLaughlin procedure, autograft or allograft reconstruction of the reverse Hill-Sachs lesion [5, 15, 16] may be inevitable. Nonoperative treatment, supervised neglect, is accepted in patients with medical high risk for surgery and uncontrollable seizure disease and elderly patients with limited demand and normal motion of contralateral glenohumeral joint [5, 15].

In conclusion, posterior dislocation of the shoulder is a usual serious misdiagnosis. The best way for preventing unrecognized cases is suspicious in patients with pain and limitation of external rotation particularly if they have history of seizure, electrical shock, or significant trauma. CT scan can help to diagnose it early. Closed reduction of posterior shoulder dislocation in cases with medium defect in the humeral head articular surface is a suitable strategy for cooperative athlete-body patients.
